# Identification of a novel heterozygous missense *TP63* variant in a Chinese pedigree with split-hand/foot malformation

**DOI:** 10.1186/s12920-022-01311-y

**Published:** 2022-07-13

**Authors:** Mingzhu Miao, Shoulian Lu, Xiao Sun, Meng Zhao, Jue Wang, Xiaotan Su, Bai Jin, Lizhou Sun

**Affiliations:** 1 Department of Obstetrics and Gynecology, The First Affiliated Hospital of Nanjing Medical University, Nanjing, 210029 Jiangsu China; 2 Department of Radiology, The First Affiliated Hospital of Nanjing Medical University, Nanjing, 210029 Jiangsu China; 3 Department of Bioinformatics, Berry Genomics Co., Ltd., Beijing, China

**Keywords:** Split-hand/foot malformation, *TP63*, Missense variant, Genetic counseling, Prenatal care

## Abstract

**Background:**

Tumor protein p63 is an important transcription factor regulating epithelial morphogenesis. Variants associated with the *TP63* gene are known to cause multiple disorders. In this study, we determined the genetic cause of split-hand/foot malformation in a Chinese pedigree.

**Methods:**

For this study, we have recruited a Chinese family and collected samples from affected and normal individuals of the family (three affected and two normal). Whole exome sequencing was performed to detect the underlying genetic defect in this family. The potential variant was validated using the Sanger sequencing approach.

**Results:**

Using whole-exome and Sanger sequencing, we identified a novel heterozygous pathogenic missense variant in *TP63* (NM_003722.5: c.921G > T; p.Met307Ile). This variant resulted in the substitution of methionine with isoleucine. Structural analysis suggested a resulting change in the structure of a key functional domain of the p63 protein.

**Conclusion:**

This novel missense variant expands the *TP63* variant spectrum and provides a basis for genetic counseling and prenatal diagnosis of families with split-hand/foot malformation or other *TP63*-related diseases.

**Supplementary Information:**

The online version contains supplementary material available at 10.1186/s12920-022-01311-y.

## Background

Split-hand/foot malformation (SHFM) is a severe congenital acral deformity characterized by the absence or hypoplasia of the central axis of the hand and foot, resulting in congenital limb dysplasia with varying degrees of fusion of the residual digit [1]. SHFM can be isolated or syndromic [2]. SHFM phenotypes are highly heterogeneous and present with variable severity, which can differ significantly even among patients from the same family [3]. Mild phenotypes are primarily characterized by single syndactyly, severe phenotypes by lobster-like or chelate-shaped hands and feet, and the most severe phenotypes by monodactyly [4]. The prevalence of SHFM ranges from 1/6000 to 1/20000; its incidence in China is the highest in the world at 1.64/10000 [5, 6].

The pathogenesis of SHFM is influenced by multiple loci and genetic patterns. Six different gene loci have been implicated in SHFM: SHFM1 (7q21–q22), SHFM2 (Xq26), SHFM3 (10q24), SHFM4 (3q28), SHFM5 (2q31), and SHFM6 (12q13) [7]. SHFM4 is an autosomal dominant genetic disease caused by variants in the gene *TP63*, which is located on chromosome 3q28 and encodes the tumor protein p63 (Online Mendelian Inheritance in Man (OMIM) entry *603273) [8]. p63 is a transcription factor of the p53 family. The p63 protein plays important roles in embryonic development and ectodermal cell differentiation [9]. In addition, it is a critical regulator of the development of the apical ectodermal ridge (AER) of limb buds [10]. The AER is a transient multilayered ectoderm that serves as a signal center essential for the development of the distal limb; a failure to maintain the AER can cause limb dysplasia. Moreover, p63 has been shown to be essential for maintaining the populations of progenitor cells necessary for epithelial development and morphogenesis [11]. To date, 148 *TP63* variants have been identified; however, only 15 have been associated with SHFM4, accounting for approximately 10% of all variants [12].

This study focuses on a Chinese family with SHFM4. The proband was a fetus with diagnosed SHFM by ultrasound, which led to an induced pregnancy termination. The underlying genetic defect in this family was detected using whole-exome sequencing (WES). A novel missense variant of *TP63* was identified and associated with SHFM4. This study expands the variant spectrum of *TP63* and sheds light on the importance of genetic counseling and prenatal diagnosis.

## Methods

### Subjects

**Fig. 1:**
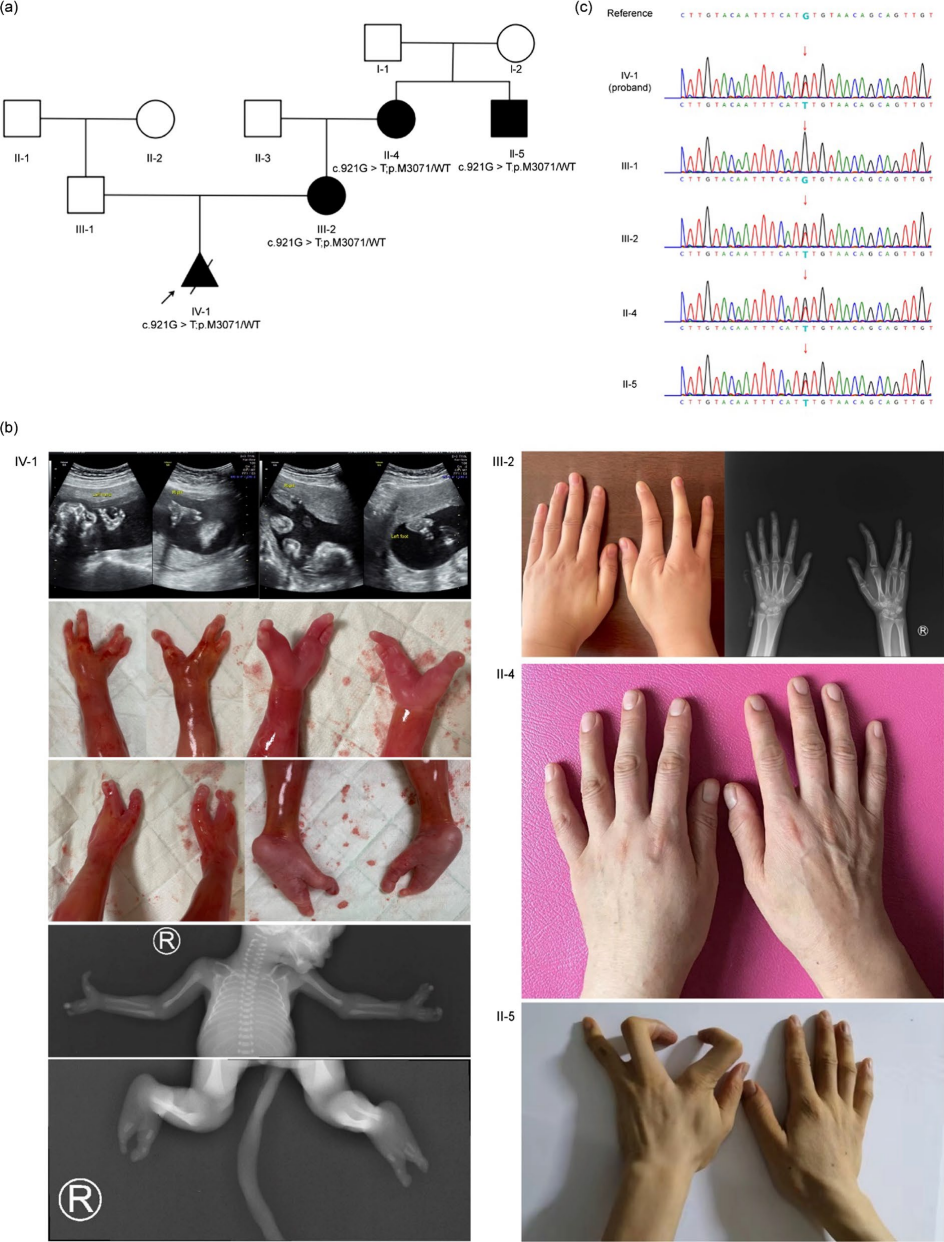
Family pedigree, clinical phenotype, and Sanger sequencing results of individuals who were subjected to WES. **a** A consanguineous pedigree showing four affected members (II-4, II-5, III-2, and IV-1) in the four-generation family. **b** Clinical features of the affected individuals, II-4, II-5, III-2, and IV-1. **c** The Sanger sequencing results of II-4, II-5, III-1, III-2, and IV-1. The red arrows indicated the substitution

The consanguineous pedigree with three SHFM affected members was recruited (Fig. 1a). The proband’s mother (III-2) was treated at the First Affiliated Hospital of Nanjing Medical University in May 2021. The proband (IV-1) was a fetus diagnosed with SHFM by prenatal ultrasound. WES was performed on three affected (II-5, III-2, and IV-1) and two unaffected (II-4 and III-1) individuals of this family.

### Whole exome sequencing and bioinformatic analysis

With the consent of the pregnant woman and her family, umbilical cord samples of the proband and peripheral blood samples from his parents, grandmother, and great uncle were collected (2–3 ml; EDTA anticoagulant). Genomic DNA was extracted using a QIAamp DNA extraction kit (Qiagen GmbH, Hilden, Germany) according to the manufacturer’s instructions.

Briefly, 1 μg genomic DNA was extracted from 200 μL peripheral blood, using a Qiagen DNA Blood Midi/Mini kit (Qiagen GmbH, Hilden, Germany) following the manufacturer’s protocol. DNA (50 ng) was digested to yield 200-bp fragments; these fragments were end-repaired, and a 3’ adenine was added. The fragments were ligated to barcoded sequencing adaptors, after which fragments of approximately 320 bp were collected using XP beads (Berry Genomics, Beijing, China). After polymerase chain reaction (PCR) amplification, DNA fragments were hybridized and captured using Berry’s NanoWES Human Exome V1.0 (Berry Genomics, Beijing, China) according to the manufacturer’s protocol. The hybrids were eluted, collected, subjected to PCR amplification, and purified. Libraries were quantified by real-time PCR and size distribution was determined using an Agilent Bioanalyzer 2100 (Agilent Technologies, Santa Clara, CA, USA).

Liquid-phase probe hybridization technique was used to capture the target gene, and the NanoWES V1.0 probe (Berry Genomics, Beijing, China) was used to capture exons upstream and downstream, as well as some intronic regions with reported pathogenic variants. The Novaseq6000 platform (Illumina, San Diego, USA) with 150 bp paired-end sequencing was used to sequence the genomic DNA of the family. Raw image files were processed using CASAVA v1.82 (Berry Genomics, Beijing, China) for base calling and generating raw data. Bases with more than 85% data quality reached the Q30 (≥ Q30) standard and those with more than 95% data quality reached the Q20 (≥ Q20) standard. The rate of duplication was < 30%.

Sequencing reads were aligned to the human reference genome (hg38/GRCh38) using the Burrows–Wheeler Aligner tool [13]. PCR duplicates were removed using Picard v1.57 (http://picard.sourceforge.net/). Verita Trekker® Variants Detection System (Berry Genomics, Beijing, China) and the third-party software GATK (https://software.broadinstitute.org/gatk/) were employed for variant calling. Variant annotation and interpretation were conducted using ANNOVAR [14] and the Enliven® Variants Annotation Interpretation System authorized by Berry Genomics. The annotation databases used included: (i) human population databases, such as gnomAD (http://gnomad.broadinstitute.org/), the 1000 Genome Project (http://browser.1000genomes.org), and dbSNP (http://www.ncbi.nlm.nih.gov/snp); (ii) in silico prediction algorithms, such as SIFT (http://sift.jcvi.org), FATHMM (http://fathmm.biocompute.org.uk), Mutation Assessor (http://mutationassessor.org), CADD (http://cadd.gs.washington.edu), and SPIDEX [15]; (iii) disease and phenotype databases, such as OMIM (http://www.omim.org), ClinVar (http://www.ncbi.nlm.nih.gov/clinvar), HGMD (http://www.hgmd.org), and HPO (https://hpo.jax.org/app/); and (iv) burden analysis of missense variants databases, such as the ExAC dataset (https://exac.broadinstitute.org/), DECIPHER (https://www.deciphergenomics.org/), and PER viewer (https://per.broadinstitute.org/).

Variants were classified into five categories–pathogenic, likely pathogenic, uncertain significance, likely benign, and benign–as per the American College of Medical Genetics and Genomics (ACMG) guidelines for the interpretation of genetic variants [16]. Variants with minor allele frequencies < 1% in exonic regions or with a splicing impact were interpreted in greater depth by considering the ACMG category, evidence of pathogenicity, clinical synopsis, and inheritance model of the associated disease. For trio analysis, potential monogenetic inheritance patterns, including de novo, autosomal recessive, autosomal dominant, X-linked recessive, mitochondrial, and, where possible, imprinted gene variation, were analyzed. Full penetrance was assumed for potentially causal variants. Variants present in parents or that had been recorded in any of the above-mentioned databases or in our in-house control exomes were excluded as etiology. Once a variant was considered as the etiology of a recessive disorder, manual inspection for coverage and additional variants of the entire coding domain was undertaken using the Integrated Genomics Viewer [17, 18].

### Sanger sequencing

The variant in the *TP63* gene identified using WES (NM_003722.5: c.921G > T; p. p.Met307Ile) was confirmed by Sanger sequencing and co‑segregation analysis. Sanger sequencing was conducted using an ABI 3730XL DNA Sequencer (Applied Biosystems, Thermo Fisher Scientific, Waltham, MA, USA). Sequences were aligned to the reference sequence using Mutation Surveyor software [19, 20].

### Protein modeling

To evaluate the deleterious effects of candidate variant, we used AlphaFold DB (AF-Q9H3D4-F1) as p63 protein structure model (https://www.ebi.ac.uk/pdbe/pdbe-kb/proteins/Q9H3D4/structures). Meanwhile, we used PDB ID 3us0 as structure model to evaluate the effect of Met307 variant.

## Results

### Clinical phenotype

The proband was a fetus whose mother’s labor was induced at 22 weeks gestation because of a prenatal diagnosis of SHFM by ultrasound (Fig. 1a). The induced fetus was a male with severe SHFM (Fig. 1b, IV-1). Radiographs and photos of the proband showed syndactyly of 1st and 2nd fingers, missing 3rd finger in the left hand. The right hand showed fusion of the 1st and 2nd metacarpals and missing partial distal phalange indicting syndactyly of 1st and 2nd fingers, missing 3rd finger, and aplasia of 4th and 5th phalanges. Both feet showed as “lobster-like foot”, with missing 2nd and 3rd toes and metatarsal bones, syndactyly of 4th and 5th toes in the left foot, and with missing 2nd, 3rd and 4th toes and metatarsal bones in the right foot. (Fig. 1b, IV-1). The proband’s mother showed missing 3rd phalanx and clinodactyly of the index finger in the right hand (Fig. 1b, III-2), but her left hand and both feet were normal. The great uncle of the proband had missing 3rd finger and camptodactyly of 2nd and 4th fingers in his left hand, but a normal right hand and both feet (Fig. 1b, II-5). The father and grandmother of the proband had normal phenotypes (Fig. 1b, III-1and Fig. 1b, II-4, respectively). The phenotypes of other members in the pedigree were normal. Regrettably, the individuals II-4 and II-5 refused to take a radiograph image. We cannot search for a minimal manifestation of SHFM, which cannot be excluded in the proband’s grandmother.

### Identification and analysis of the *TP63* variant

WES was performed on five individuals of the family (II-4, II-5, III-1, III-2, and IV-1). A total of > 98% of the targeted regions were covered with a depth of more than 20 ×. After filtering whole exome data (detailed whole exome filtered data shown in Additional file 1), one candidate variant matched with known phenotype in *TP63* gene was subsequently extracted. A novel heterozygous variant in *TP63* (NM_003722.5: c.921G > T; p.Met307Ile) was identified in the proband (Fig. 1c).Sanger sequencing further revealed that the variant was identified in the proband’s mother, grandmother, and great uncle, respectively (Fig. 1c). However, the proband’s father (III-1) was unaffected, and Sanger sequencing showed he did not have the variant (Fig. 1c).

**Fig. 2:**
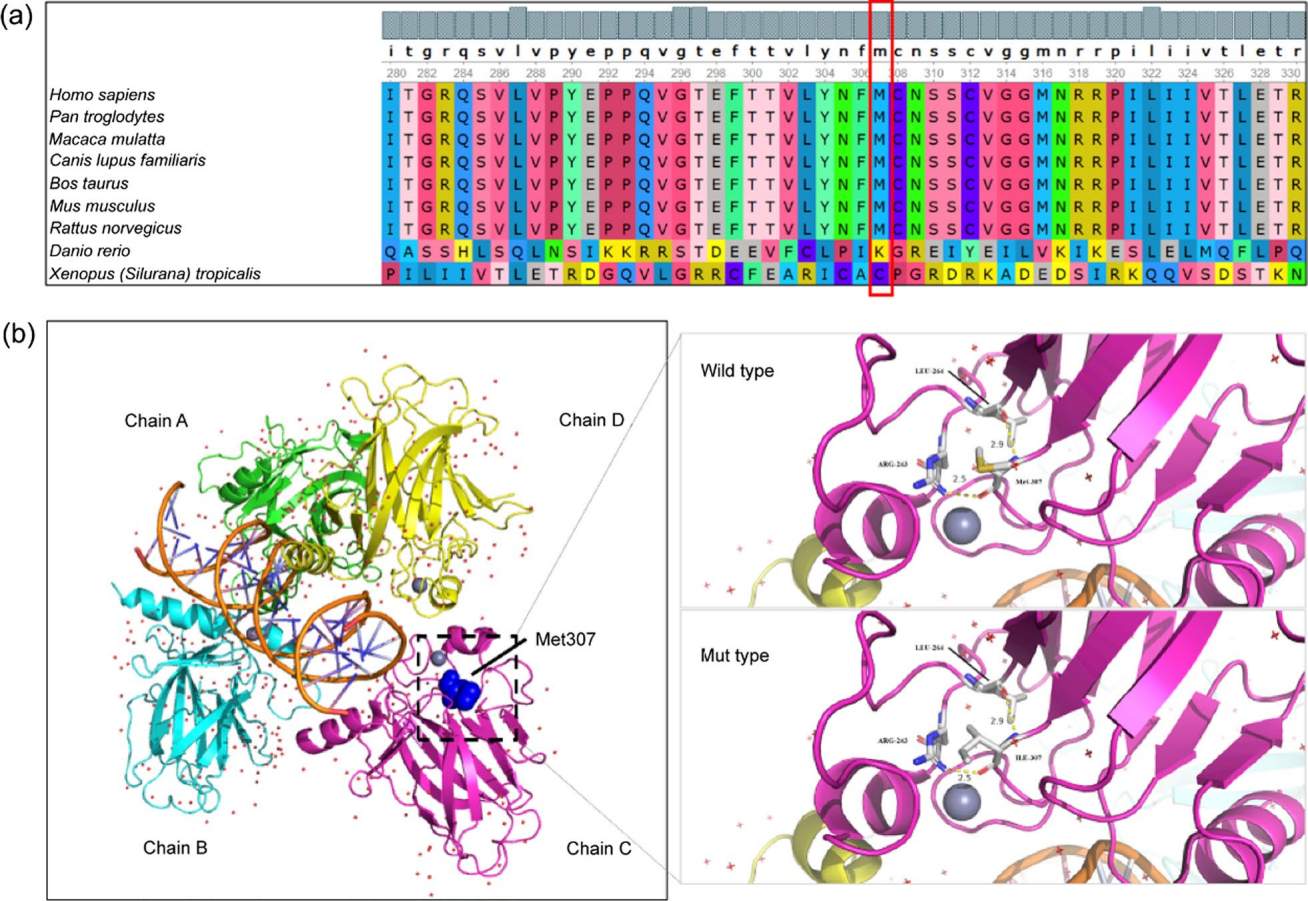
The novel variant among the species and the p63 protein structure model. **a** The Met307Ile substitution is located in a highly conserved site among vertebrates. The red box represents the mutated residue; **b** PDB ID 3us0 was used as the structural model to evaluate the effect of the Met307 variant. Stick models show the side chains of the amino acids around Met307. In wild type p63, Met307 forms two hydrogen bonds (represented by the yellow dotted lines) with Arg243 and Leu264. The hydrogen bonds are not affected in the mutant type p63

This variant was not present in the gnomAD, 1000G, and ExAC databases (PM2). It was predicted to be “disease-causing/probably damaging” by MutationTaster, REVEL, SIFT, CADD, and PROVEAN (PP3) [21–25] (Table 1). A p63 sequence alignment from multiple species showed that Met307 is highly conserved, suggesting that this residue plays a vital role in maintaining protein stability and function (Fig. 2a). Burden analysis of missense variants revealed that the variant was located in the critical functional domain (PM1). This variant segregated with the disorder in this family (PP1). According to the ACMG guidelines for the interpretation of sequence variants [16], c.921G > T (p.Met307Ile) was assessed to be likely pathogenic (PM1, PM2, PP1, PP3).

**Table 1:** The pathogenicity of the *TP63* variant

Genomic position(Hg38)	Chr3:189,867,871
cDNA change(NM_003722.5)	c.921G > T
Protein change	p.Met307Ile
Inheritance	Maternal
SIFT	Damaging (0.005)
Polyphen-2_HDIV	Benign (0.357)
Polyphen-2_HVAR	Possibly damaging (0.625)
Mutation Taster	Disease-causing (1.0)
CADD	Damaging (28.2)
GERP + +	Conserved (5.61)
REVEL	Damaging (0.827)
PROVEAN	Damaging (-3.57)

### Structure–function correlations of the *TP63* variant

PDB ID 3us0 was used as structure model to evaluate the effect of Met307 variant. As shown in Fig. 2b, residue Met307 forms two hydrogen bonds with Arg243 and Leu264 at distances of 2.5 Å and 2.9 Å, respectively. When Met307 was replaced with isoleucine, the variant did not cause a change in the hydrogen bond network (Fig. 2b), suggesting that its disruption was not the pathogenic mechanism.

## Discussion

All patients with the phenotype of SHFM had the same variant of *TP63* in this pedigree. It has been reported that heterozygous pathogenic variants in *TP63* are associated with a total of seven diseases: ectrodactyly, ectodermal dysplasia, and cleft lip/palate syndrome 3 (EEC3; OMIM #604292); split-hand/foot malformation 4 (SHFM4; OMIM #605289); ankyloblepharon-ectodermal defects-clefting syndrome (AEC; OMIM #106260); acro-dermato-ungual-lacrimal-tooth syndrome (ADULT syndrome; OMIM #103285); limb-mammary syndrome (LMS; OMIM #603543); Rapp-Hodgkin syndrome (RHS; OMIM #129400); and orofacial cleft 8 (OFC8; OMIM #618149) [11]. The phenotypes of *TP63*-related diseases vary greatly; indeed, phenotypic variability within each disease is also considerable. In cases of SHFM, the hands and feet often exhibit ectrodactyly, syndactyly, a central cleft, and aplasia of the phalanges, metacarpals, and metatarsals. Although SHFM is predominantly syndromic, it can be isolated; in these cases, it is called SHFM4, referring to a “pure” limb dysplasia without other malformations. The patients in our study showed features consistent with this condition and were therefore classified as having SHFM4.

Variants in multiple regions of the *TP63* gene are responsible for these conditions and affect the function of p63 in different ways. There is a clear genotype–phenotype correlation for *TP63* gene variants. In EEC3 and AEC syndromes, variants are clustered in the DNA binding domain (DBD), the sterile alpha motif, and/or the transactivation inhibitory domains. Variants causing SHFM4 are distributed throughout the *TP63* gene, including the transactivation and transactivation inhibitory domains, the splice site, and the DBD [8, 26, 27]; however, it remains unclear how these widely dispersed variants cause limb defects. Some variants in SHFM4 have been reported to alter the activation and stability of p63 [28, 29]. It is possible that SHFM is caused by altered p63 degradation, although different protein degradation pathways are involved [10, 28, 30]. The altered residues in the disease-causing alleles of SHFM4 appear to maintain the overall domain structure, unlike those that cause EEC3, which directly interact with DNA.

**Fig. 3:**
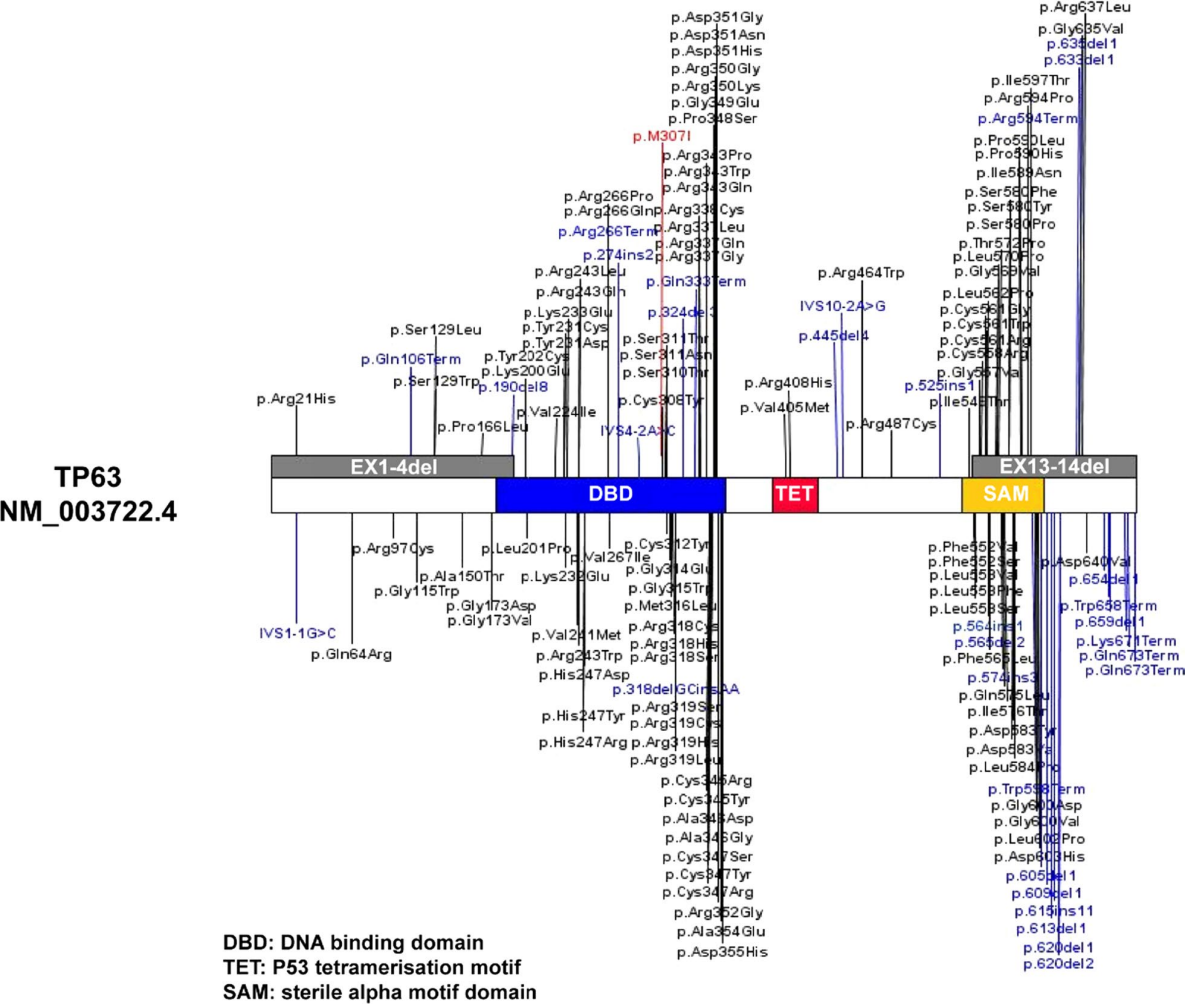
Schematic of the TAp63‐alpha protein (NM_003722.4) and all reported variants. The rectangular box represents the TAp63‐alpha protein with the N‐terminus on the left and the C‐terminus on the right. Known functional domains include the transactivation, DNA binding, oligomerization, sterile alpha motif, and transactivation inhibition domains. Blue font, null variants; black font, missense variants; red font, the variant identified in our patients

The variant in our patient (c.921G > T; p.Met307Ile) lies within the DBD of p63 (Fig. 3), which is responsible for establishing DNA interactions [8, 31]. The variant identified here is present on all protein coding isoforms. This residue is highly conserved across species (Fig. 2a). According to Grantham scores, the physicochemical differences between methionine and isoleucine are small; this new variant may cannot affect the structure of the DBD of p63. Nevertheless, this novel variant was predicted to would be disease-causing in our study. Importantly, 3us0 structure of p63 DBD is complex with a 22 base pair A/T rich response element. This structure can check the effect of the variant. The variant affects DNA binding or protein interaction ability of *TP63*. This may, in turn, affect the formation and differentiation of the AER, possibly leading to limb dysplasia.

Of the 148 variants in *TP63* identified to date (Fig. 3), more than 110 are associated with EEC3 and/or AEC syndrome [32, 33] and only 15 have been implicated in SHFM4 [12]. Despite the high phenotypic heterogeneity of SHFM, there is no clear literature or database reporting the proportion of *TP63* variants. Additionally, there is no clear report of *TP63* penetrance, although incomplete penetrance is reported for many diseases involving this gene, including SHFM4 [8, 34–37]. The incomplete penetrance in the grandmother (II-4) stays in line with similar reported cases.

Incomplete penetrance of phenotypic features and wide clinical variability are often seen within families sharing the same *TP63* variant. Therefore, it is possible that some affected individuals have mild or nearly undetectable abnormalities [38]. Co-segregation analysis revealed, that the variant was present in 4 individuals of the examined family of whom only 3 were affected with the SHFM4. Despite carrying the gene variant, II-4 showed no symptoms of SHFM4, indicating that this variant may have incomplete penetrance. Incomplete penetrance for SHFM4 has been reported [8, 36]. Incomplete penetrance has also been observed for other *TP63*-related disorders, including ADULT syndrome [34, 35], EEC3 [8], and orofacial cleft 8 [37, 39]. *p63* knockout mice(*p63*^−/−^) have striking developmental defects, and heterozygous *p63* knockout mice (*p63*^+/−^) do not display ectodermal defects [11, 40]. However, heterozygous *p63*^+/EEC^ mice generated by knocking in the R279H allele rarely display ectrodactyly [41], whereas *p63*^EEC/EEC^ mice resemble *p63*^−/−^ mice. The development of EEC in patients carrying this mutated allele [12] indicates that mouse models react somewhat differently from humans to disturbed balances of wild-type versus mutated p63 molecules. This difference in response could be due to differences in the stability of the mutated mouse and human p63 proteins [42]. There was not incomplete penetrance reported in mouse *TP63* model.

Clinical spectrum of the SHFM differs from patient to patient in severity and even varies among individuals in the same family [3, 43]. The proband has bilateral SHFM while his mother and great uncle have unilateral split hand. The phenotypic variability in this family was consistent with those reported in previous studies [8, 36]. This variable phenotype within family suggests the involvement of a modifier allele that contribute the complete penetrance and full expression of the phenotype in the proband. Perhaps a polymorphism within the *TP63* gene itself is responsible for this effect [44]. Just as reported, the absence of ectodermal phenotypes in patients segregating previously reported variant in *TP63* highlights the importance of modifier genes causing variations in *TP63*-related SHFM phenotypes [45]. The phenotypic differences depend on variability of *TP63* expression [46]. Further research is warranted to determine whether these findings are attributable to epigenetic processes or to genetic modifiers. In-depth studies are needed to explore the specific pathogenesis.

SHFM4 is inherited in an autosomal dominant manner. Each child of an individual with SHFM4 has a 50% chance of inheriting the pathogenic variant. In the absence of functional data, a variant with incomplete penetrance identified in this study should be interpreted cautiously for prenatal diagnosis. It is therefore reasonable to recommend that pregnancy monitoring by non-invasive ultrasound should be offered to all first degree relatives of an individual with the variant. Prenatal diagnosis for pregnancies diagnosed SHFM by ultrasound is recommended for molecular genetic testing of *TP63* [47]. Differences in perspective may exist among medical professionals and within families regarding the use of prenatal testing, particularly if the testing is being considered for the purpose of pregnancy termination rather than early diagnosis. While most centers would consider decisions regarding prenatal testing to be the choice of the parents, discussion of these issues is appropriate. It is appropriate to offer genetic counseling (including discussion of potential risks to offspring and reproductive options) to young adults who are affected or at risk [47].

## Conclusion

In this study, we presented a novel heterozygous missense variant (NM_003722.5: c.921G > T; p.Met307Ile) of *TP63* identified by WES in a Chinese family with SHFM. This study provides insights that will contribute to our understanding of this new variant of the *TP63* gene, which is likely to be pathogenic. Our findings shed light on the importance of genetic counseling and prenatal diagnosis (Additional file 1).

## Supplementary Information

**Additional file 1.** Detailed whole exome filtered data.

## Data Availability

The datasets analyzed during the current study are available from the NCBI SRA under the accession number PRJNA832422 (https://www.ncbi.nlm.nih.gov/sra/?term=PRJNA832422).
